# Imaging features of glucagonoma syndrome: A case report and review of the literature

**DOI:** 10.3892/ol.2015.2930

**Published:** 2015-02-03

**Authors:** WEI-FU LV, JIAN-KUI HAN, XIN LIU, SHI-CUN WANG, BO PAN, AO XU

**Affiliations:** 1 Positron Emission Tomography/Computed Tomography Center, Qilu Hospital, First Affiliated Hospital of Shandong University, Jinan, Shandong 250012, P.R. China; 2 Department of Radiology, Affiliated Anhui Provincial Hospital of Anhui Medical University, Hefei, Anhui 230001, P.R. China; 3 Positron Emission Tomography/Computed Tomography Center, Affiliated Anhui Provincial Hospital of Anhui Medical University, Hefei, Anhui 230001, P.R. China; 4 Department of Pathology, Affiliated Anhui Provincial Hospital of Anhui Medical University, Hefei, Anhui 230001, P.R. China

**Keywords:** hyperglycemia, glucagon, pancreas, neuroendocrine tumour, imageology

## Abstract

Glucagonoma syndrome appears as an extremely rare neuroendocrine tumour, with few studies ever having detailed its imaging manifestations. In particular, the magnetic resonance imaging (MRI) features of the lesion have not yet been reported. The present study describes a 54-year-old male who presented with uncontrollable skin erythema and weight loss that had been apparent for two years, and diabetes mellitus that had been apparent for five years. The glucagon level was 180 pg/ml. The plain abdominal computed tomography (CT) scan revealed a solid tumour in the neck of the pancreas, which was slightly reinforced during the arterial phase of the enhanced CT scan. Upon MRI, the lesion exhibited a low signal on T1-weighted imaging, and a slightly high signal on T2-weighted and half-Fourier acquisition single-shot turbo spin echo sequence imaging, which measured ~4.5×3.0×3.0 cm in size. Upon diffusion-weighted imaging, the lesion demonstrated heterogeneous hyperintensity, which was mildly enhanced during the arterial phase and washed out during the portal venous phase of gadopentetate dimeglumine-enhanced MRI. ^18^F-fludeoxyglucose (^18^F-FDG) positron emission tomography (PET)-CT identified a mild uptake of ^18^F-FDG by the lesion. The patient was diagnosed with glucagonoma syndrome, and a distal pancreatectomy and splenectomy were subsequently performed. Microscopy revealed that the tumour cells exhibited nest- and belt-like arrangements. The immunohistochemical staining identified positive reactions for glucagon, synaptophysin and chromogranin A, which are consistent with a diagnosis of glucagonoma. Following surgery, the symptoms disappeared and the glucagon level returned to normal. In conclusion, imaging examinations are useful for determining the location and size of a glucagonoma. In particular, MRI is able to identify the distinctive morphological features of the lesion. Immunohistochemical staining provides diagnostic evidence based upon the neuroendocrine features.

## Introduction

Glucagonoma is an extremely rare neuroendocrine tumour that accounts for 1% of neuroendocrine tumours and <5% of all primary pancreatic malignancies (1,2). Although glucagonoma may appear as a benign neoplasia, at least 50% of glucagonomas cause metastatic disease when diagnosed (2). If the disorder is complicated with systemic clinical manifestations, including necrolytic migratory erythema, hyperglucagonaemia, diabetes mellitus, anaemia, weight loss, glossitis, cheilitis, steatorrhoea, diarrhoea, venous thrombosis and neuropsychiatric disturbances, it is referred to as glucagonoma syndrome (3). At present, the standard treatment for glucagonoma syndrome is surgical resection (2). The early and accurate diagnosis of this syndrome may lead to positive treatment outcomes and an improved prognosis. However, few studies have detailed the imaging features of glucagonoma (4). In particular, the magnetic resonance imaging (MRI) features of the lesion have not yet been reported. A male with glucagonoma syndrome was admitted to the First Affiliated Hospital of Shandong University (Jinan, China). Imaging modalities, including computed tomography (CT), MRI and ^18^F-fludeoxyglucose (^18^F-FDG) positron emission tomography (PET)-CT were performed, and imaging findings were characterised. The present study describes and discusses these imaging features.

## Case report

A 54-year-old male was admitted to the First Affiliated Hospital of Shandong University (Jinan, China) due to persistent and progressive skin eruptions and weight loss of ~20 kg over the past two years. The medical history revealed that the patient had previously been diagnosed with Behçet’s disease and treated with corticosteroids during multiple hospital admissions. However, there had been no significant improvement of the symptoms. In addition, the patient had suffered with diabetes mellitus for five years. The patient stated that the skin erythema had recently become progressively worse. The family history was negative for multiple endocrine neoplasia and diabetes mellitus. Upon physical examination, the patient exhibited cyclical itchy skin lesions on the face, back, groin and lower limbs. The centres of the lesions were hypopigmented or slightly scaly. There was no palpable mass evident in the abdomen.

Laboratory analysis revealed normocytic anaemia [red blood cell count, 2.59×10^12^/l (normal range, 4.0–5.0×10^12^/l); haemoglobin level, 81 g/l (normal range, 120–160 g/l); mean corpuscular volume, 86.1 fl (nromal range, 80–100 fl); mean corpuscular haemoglobin level, 29.5 pg (normal range, 27–33 pg); and mean corpuscular haemoglobin concentration, 343 g/l (normal range, 320–360 g/l)], hyperglucagonaemia (181.00 pg/ml; normal range, 50–50 pg/ml) and hyperglycaemia (fasting blood glucose level, 165.6 mg/dl; normal range, 70–100 mg/dl). In addition, the expression of the tumour marker, carbohydrate antigen 19–9, was markedly increased (180.10 U/ml; normal range, 0–37 U/ml), whereas the α-fetoprotein and carcinoembryonic antigen levels were within the normal ranges. The exocrine function was also normal.

**Figure 1:**
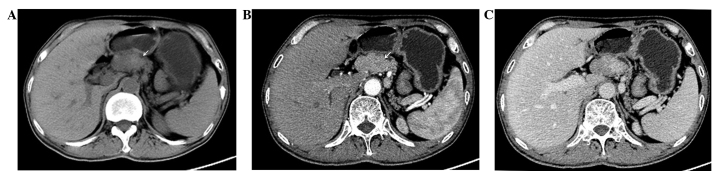
Computed tomography (CT) manifestations of glucagonoma syndrome. (A) Plain abdominal CT revealing an obscure mass (arrow) in the neck of the pancreas. The lesion was slightly enhanced during (B) the arterial phase and washed out during (C) the portal venous phase upon enhanced CT.

**Figure 2:**
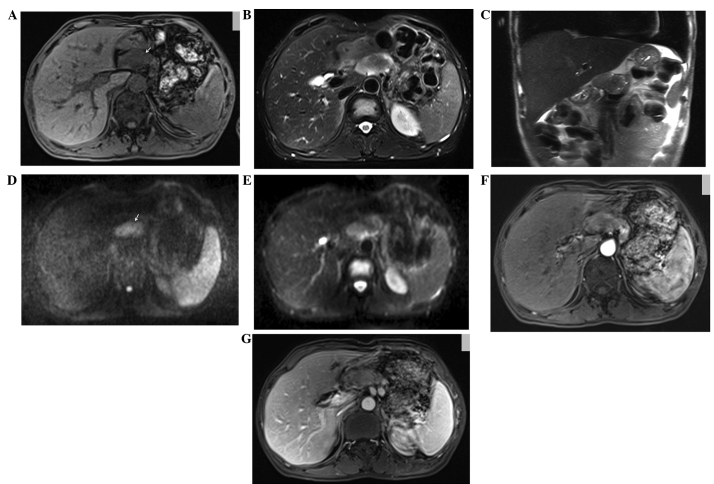
Magnetic resonance imaging manifestations of glucagonoma syndrome. The lesion exhibited a low signal intensity (arrow) on (A) T1-weighted imaging (WI), and a slightly high signal intensity on (B) T2WI (with fat-suppression) and (C) half-Fourier acquisition single-shot turbo spin echo sequence imaging. Diffusion-WI revealing the presence of a lesion with (D and E) heterogeneous hyperintensity (arrow), which was mildly reinforced during (F) the arterial phase and washed out during (G) the portal venous phase during enhanced imaging.

**Figure 3:**
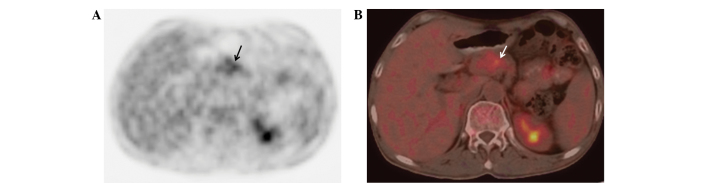
Positron emission tomography-computed tomography (PET-CT) manifestations of glucagonoma syndrome. (A and B) PET-CT revealing the presence of a lesion with mild ^18^F-fludeoxyglucose uptake (arrow), which corresponds to the location of the tumour identified by CT and magnetic resonance imaging.

The plain abdominal CT scan identified an obscure mass in the neck of the pancreas with a vague margin. Upon enhanced CT, the lesion was slightly enhanced during the arterial phase and washed out during the portal venous phase. The body and tail of the pancreas were atrophied. There was no evidence of enlarged lymph nodes or liver metastases (Fig. 1). Upon MRI, the lesion exhibited a low signal intensity on T1-weighted imaging (WI), and a slightly high signal intensity on T2WI and half-Fourier acquisition single-shot turbo spin echo sequence imaging, which measured ~4.5×3.0×3.0 cm in size. Upon diffusion-WI (DWI), the lesion demonstrated heterogeneous hyperintensity, which was mildly reinforced during the arterial phase and washed out during the portal venous phase of gadopentetate dimeglumine-enhanced imaging (Fig. 2). The ^18^F-FDG PET-CT revealed mild ^18^F-FDG uptake by the lesion (standardised uptake value, 3.8) in the neck of the pancreas, which corresponded to the location of the tumour identified by the CT and MRI scans (Fig. 3).

**Figure 4:**
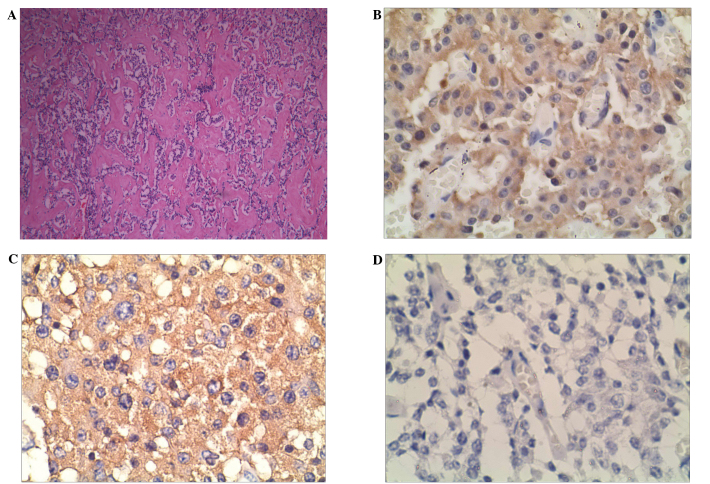
Histopathological and immunohistochemical staining features. (A) Microscope analysis revealing the tumour cells in nest- and belt-like arrangments, with uniform round and polygonal tumour cells, pale cytoplasm and round nuclei (haematoxylin and eosin staining; magnification, ×20). Immunohistochemical staining revealing a positive reaction for (B) glucagon and (C) synaptophysin, and a weakly positive result for (D) insulin (magnification, ×400).

Based upon the clinical presentation and imaging findings, the patient was diagnosed with glucagonoma syndrome. A distal pancreatectomy and splenectomy were subsequently performed. The regional lymph nodes were also dissected. The histopathological examination revealed that the tumour was composed of uniform round and polygonal cells, with pale cytoplasm and round nuclei. The tumour cells exhibited nest- and belt-like arrangements. The immunohistochemical staining identified positive reactions for glucagon, synaptophysin and chromogranin A, a weakly positive reaction for insulin (Fig. 4), and negative reactions for gastrin and somatostatin. The 12 dissected regional lymph nodes were not affected.

During the eight-month post-surgery follow-up period, the skin lesions disappeared and the plasma glucagon levels returned to normal.

The present study was approved by the Institute Ethics Committee of the First Affiliated Hospital of Shandong University, and written informed consent was obtained from the patient for the publication of the study and any accompanying images.

## Discussion

Glucagonoma may appear as a benign or slow-growing metastasising malignant tumour (5,6). The first case of glucagonoma was described by Becker *et al* (7) in 1942, in which the patient suffered from pancreatic neoplasia complicated with skin erythema, diabetes mellitus and anaemia.

In the present study, the CT scans identified an obscure mass in the head and neck of the pancreas, but no distinctive features. However, the MRI scans using T1WI or T2WI identified morphological characteristics, including the contour and internal structures of the lesion. DWI is extremely sensitive to the motion of water protons at the microscopic level in response to thermal energy (8). In contrast to the low signal intensity of the normal pancreas, pancreatic tumours may exhibit high signal intensities (8). Upon DWI, the present case demonstrated certain features typical of a tumour.

Teixeira *et al* (5) reported that glucagonomas demonstrate significant hypervascularity, and that selective celiac and superior mesenteric arteriographies were the most reliable ways to detect the primary neoplasm. Although selective arteriographies were not performed in the present study, the lesion exhibited slight enhancement upon enhanced CT and MRI, which was not a result of hypervascularity. These findings are not consistent with the previous literature, and therefore suggest that glucagonoma may exhibit additional haemodynamic patterns.

PET-CT remains as a promising imaging technique for detecting the presence of tumours. ^18^F-FDG is considered to be a tracer of glucose metabolism, as its molecular structure is similar to that of glucose. Subsequent to injection, ^18^F-FDG can be transported into the cell through the glucose transporter proteins on the cell membrane. Consequently, PET-CT identifies high ^18^F-FDG uptake in rapidly growing tumours, in which the glycolysis rate is increased (9). In the present study, the tumour exhibited mildly increased ^18^F-FDG uptake, and no metastases were detected in any other organs.

Stacpoole (10) stated that the following criteria should be fulfilled in order to diagnose glucagonoma syndrome: i) Detection of a tumour by direct visualisation or imaging examination; ii) evidence that the tumour demonstrates a preponderance of glucagon-containing cells; iii) an increase in the level of basal circulating immunoreactive glucagon; and iv) the presence of a skin rash, glucose intolerance and hypoaminoacidaemia, alone or in combination. In the present study, the results of the microscopic examination were consistent with the features of a neuroendocrine neoplasm, and the immunohistochemical staining was positive for glucagon, synaptophysin and chromogranin A. These findings confirmed the diagnosis of a glucagonoma.

Surgical resection is the optimal strategy for the treatment of glucagonoma (11). Depending on the location, size and pathological type of the tumour, the surgical approach can be divided into local resection, pancreatoduodenectomy, or pancreatic body and tail resection (12). In the present study, the skin lesions disappeared and the plasma glucagon levels returned to normal shortly after the surgery.

In conclusion, glucagonoma syndrome exhibits certain typical clinical manifestations. Imaging examinations are useful for determining the location and size of a glucagonoma, and in particular, MRI can identify distinctive morphological features. Immunohistochemical analysis provides diagnostic evidence based upon the neuroendocrine features. The present study summarized the multimodality imaging features of glucagonoma, which are of great importance for the differential diagnosis of pancreatic tumours.
